# Zinc Ion Hybrid Capacitors: Four Essential Parameters Determining Device Energy Density

**DOI:** 10.1002/advs.202408997

**Published:** 2024-11-06

**Authors:** Jiacheng Wu, Di Zhu, Yuqi Pan, Justin Prabowo, Li Wei, Yuan Chen

**Affiliations:** ^1^ School of Chemical and Biomolecular Engineering The University of Sydney Darlington NSW 2006 Australia

**Keywords:** energy density, zinc ion hybrid capacitor

## Abstract

Zinc ion hybrid capacitors (ZIHCs) with Zn metal faradic and carbon capacitive electrodes have potential applications in grid‐scale energy storage systems and wearable devices. However, the high specific energy density reported in many recent studies is based on the mass of active carbon materials alone, with deficient device energy density. This perspective article discusses how four crucial parameters influence the device energy density of ZIHCs, including areal mass loading (*m_c_*) and specific capacity (*Q*_*g*,*c*_) of active carbon materials in cathodes, negative‐to‐positive electrode capacity ratio (N/P), and electrolyte‐to‐active carbon materials mass ratio (E/C). Using a representative device model, how the device energy density varies when these four parameters change is shown. Detailed analysis indicates that specific parameter windows with the four parameters within narrow ranges (e.g., *m_c_* = 10–20 mg cm^−2^, *Q*_*g*,*c*_ > 100 mAh g^−1^, N/P < 20, and E/C < 5) need to be achieved simultaneously to deliver application‐relevant energy density (e.g., >30 Wh kg^−1^) in ZIHCs. It is hoped that these findings assist in objectively evaluating reported performance data and identifying essential issues for future research development to realize practical applications.

## Graphical abstract

Four parameters need to be achieved simultaneously within narrow ranges (e.g., *m_c_* = 10–20 mg cm^−2^, *Q*_*g*,*c*_>100 mAh g^−1^, N/P <20, and E/C <5) to deliver application‐relevant energy density (e.g., >30 Wh kg^−1^) in zinc ion hybrid capacitors.

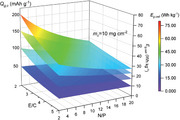

## 1 Introduction

**Figure 1:**
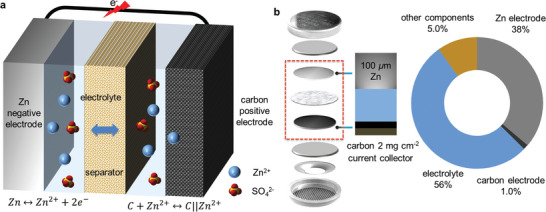
a) Schematic illustration of the structure and working mechanism of a zinc ion hybrid capacitor. b) The mass distribution of different zinc ion hybrid capacitors’ components with a coin cell structure.

Both grid‐scale energy storage systems that integrate electricity generated from renewable energy sources and energy storage units that harvest energy from body movements to power wearable electronics face intermittent and variable charging/discharging, which creates technical challenges for regular batteries.^[^ ^1^ ^]^ Supercapacitors based on ions’ reversible and fast physisorption on high‐surface‐area porous carbon materials offer fast charge/discharge rates and long cycle life, ideal for handling intermittent and variable charge/discharge.^[^ ^2^ ^]^ However, they have relatively low energy density (typically less than 10 Wh kg^−1^). Hybrid capacitors that use one capacitive electrode to enable fast charge/discharge and another battery‐like faradic electrode to increase energy storage density may deliver higher energy storage capacity while retaining supercapacitors’ fast charge/discharge rate.^[^ ^3^ ^]^ Among potential candidates, zinc ion hybrid capacitors (ZIHCs) stand out because of the high capacity of Zn (i.e., 820 mAh g^−1^), superior safety, and low cost.^[^ ^4^ ^]^ It has the potential to compete with or complement commonly used lead‐acid batteries with an energy density of 30–50 Wh kg^−1^ in grid‐scale energy storage systems and find applications in wearable devices. As illustrated in **Figure** **1**a, a typical ZIHC consists of a Zn metal foil as the faradic electrode, a carbon material capacitive electrode, a liquid or hydrogel electrolyte containing Zn salts, and an ion‐permeable separator if a liquid electrolyte is used.

Much recent research has focused on improving the specific energy storage capacity of carbon electrodes, the cycling stability of Zn electrodes, and the formulation of electrolytes to deliver high energy storage density and long cycling life.^[^ ^4b,d^ ^]^ Table S1 (Supporting Information) summarizes the energy storage performance reported in representative recent studies. The reported devices delivered an average gravimetric specific density of ≈175 mAh g^−1^ (based on the mass of active carbon materials in electrodes) at the relatively low current density ranging from 0.1 to 0.5 A g^−1^, associated with an average device energy density of ≈135 Wh kg^−1^ (also based on the mass of active carbon materials in electrodes), with a few studies achieving a gravimetric energy density beyond 200 Wh kg^−1^. Although these studies provide important insight into the working principles of ZIHCs and raise hopes for their practical applications, there is a significant gap between the results reported in current research studies and the realistic performance that could be achieved in practical devices. Considering a typical coin cell (with a surface area of 1.13 cm^2^) used in many recent studies (Figure 1b), a thick Zn electrode (i.e., 100 µm thickness) and a carbon electrode with relatively low mass loading of active materials (e.g., 2.0 mg cm^−2^) would be sandwiched between excessive electrolytes of 90 µL of 2 m ZnSO_4_ electrolyte (density of 1.31 g cm^−3^). The electrode's weight of active carbon materials only counted for 1.0 wt.% of the total device weight (see calculation details and Table S2, Supporting Information). Thus, the actual device energy density is much lower than the reported values based on the mass of active carbon materials alone.^[^ ^5^ ^]^ This gap has become a critical hurdle in objectively identifying essential issues toward realistic applications.

We propose that the practical device energy density of ZIHCs is simultaneously influenced by four critical parameters, including areal mass loading and specific capacity of active carbon materials, negative‐to‐positive electrode capacity ratio, and electrolyte‐to‐active carbon materials mass ratio. It should be noted that similar parameters’ influences on energy storage performance have also been raised in different battery systems.^[^ ^6^ ^]^ The interplay of these four parameters in ZIHCs is not straightforward, and the strategies for optimizing them are often missing in current studies of ZIHCs. This perspective article systematically analyzes their effects on the gravimetric cell energy density of ZIHCs using a representative ZIHC device model. We show that developing ZIHCs within specific windows of these four parameters is essential to deliver desirable energy density. Some strategies to improve these parameters and their potential hurdles are briefly discussed.

## 2 Critical Parameters of ZIHCs

We first define the four critical parameters and discuss their significance. The areal mass loading of active carbon materials (*m_c_*, mg cm^−2^) in a carbon material‐based positive electrode is defined as the weight of active carbon materials (*M_c_*, mg) per unit surface area of the positive electrode (*A*, cm^2^; Equation 1).^[^ ^7^ ^]^

(1) $$m_{c}=\frac{M_{c}}{A}\;$$

*m_c_* in commercial supercapacitors is usually ≈10–20 mg cm^−2^, while it is often ≈2–3 mg cm^−2^ in current ZIHC studies.^[^ ^8^ ^]^ Lower *m_c_* is used because ion transport and electrode transfer are more efficient in low‐mass‐loading electrodes. In contrast, high‐mass‐loading electrodes bring significant mass transfer resistance and higher electrical contact resistance, which causes slower charge storage behaviors.^[^ ^9^ ^]^ This is reflected by poor rate performance at higher current densities and lower specific capacity when high‐mass‐loading electrodes are used.^[^ ^5^ ^]^

Unlike metal oxide cathode materials used in batteries, active carbon materials used in supercapacitor electrodes do not possess a theoretical nominal energy storage capacity. Their charge storage capability is often described using the term “gravimetric specific capacitance” (*C*_*g*,*c*_, F g^−1^), correlating the amount of charge storage (*q*, C) within a specific voltage span (*V*, V) per weight of the active carbon materials (Equation 2).^[^ ^10^ ^]^

(2) $$\;C_{g,c}=\frac{q}{V\times \frac{M_{c}}{1000}}\;$$

*C*_*g*,*c*_ can be converted to a dummy carbon gravimetric specific capacity (*Q*_*g*,*c*_, mAh g^−1^) by Equation (3).

(3) $$\;Q_{g,c}=\frac{C_{g,c}\times 1.6}{3.6}$$

For example, commercial activated carbon (i.e., YP‐50F) used in supercapacitors shows a *Q*_*g*,*c*_ of ≈60 mAh g^−1^ in ZIHCs.^[^ ^11^ ^]^ Much higher *Q*_*g*,*c*_ has been reported for various carbon materials in recent studies (Table S1, Supporting Information), which often correlate with large specific surface area, appropriate pore size distribution between micropores and mesopores, high electrical conductivity, and the inclusion of different heteroatom dopants (i.e., O, N, S, P).^[^ ^4^, ^12^ ^]^

With the areal mass loading of active carbon materials *m_c_*, *Q*_*g*,*c*_ can be converted to the areal capacity (*Q*_*A*,*P*_, mAh cm^−2^) of carbon positive electrodes (Equation 4).^[^ ^13^ ^]^

(4) $$\;Q_{A,P}=0.001\times Q_{g,c}\times m_{c}$$

The negative‐to‐positive electrode capacity (N/P) ratio represents the areal capacity ratio between Zn negative electrodes (*Q*_*A*,*N*_, mAh cm^−2^) and carbon positive electrodes (Equation 5). The reciprocal of the N/P ratio (Equation 6), known as the depth of discharge (*DOD*, %), is often used in Zn ion battery studies to quantify the percentage of the charge stored at the cathode originating from the Zn anode, thereby indicating the utilization ratio of the Zn anode.^[^ ^8^, ^14^ ^]^

(5) $$\;\frac{N}{P}=\frac{Q_{A,N}}{Q_{A,P}}$$

(6) $$\mathrm{DOD}=\frac{1}{\frac{N}{P}}\times 100\%$$

Ideally, an N/P ratio of 1 (100% *DOD*) would balance the energy storage capacity of two electrodes, where all the stripped Zn ions from Zn electrodes are utilized for energy storage at carbon electrodes. However, as the side reactions and dendrite formation constantly consume Zn metal, additional Zn must be replenished to enable stable charge/discharge cycling, usually through a thick Zn foil, which results in a high N/P ratio (e.g., >100) and a low Zn electrode utilization ratio (e.g., *DOD* < 1%), as the case of many recent studies.^[^ ^15^ ^]^

The relative areal mass ratio between electrolytes (*m_E_*, mg cm^−2^) and the corresponding areal mass loading of active carbon materials (*m_c_*) can present the dimensionless electrolyte‐to‐carbon mass ratio (E/C) in a ZIHC (Equation 7).

(7) $$\frac{E}{C}=\frac{m_{E}}{m_{c}}$$

Although few studies have reported the exact amounts of electrolytes used (Table S1, Supporting Information), excessive electrolytes are often used to compensate for electrolyte depletion from parasitic reactions to retain stable cycling performance.^[^ ^16^ ^]^ Take the representative coin cell discussed above as an example; its E/C ratio is 52.2. If carbon materials have a high *Q*_*g*,*c*_ of 200 mAh g^−1^, this ZIHC would require ≈260 g electrolyte to deliver 1 Ah of charge storage, which is 200 times the amount needed by typical lithium‐ion batteries (e.g., 1.3 g electrolyte per Ah).^[^ ^17^ ^]^ This results in a deficient device energy density for ZIHCs.

## 3 A Representative ZIHC Model

Using a representative ZIHC model, we next systematically analyze the influences of the four critical parameters (*m_c_*, *Q*_*g*,*c*_, N/P, and E/C) on the gravimetric cell energy density (*E*_*g*,*cell*_, Wh kg^−1^). This ZIHC model is based on a typical coil cell used in current studies. However, it should be noted that we exclude the mass contributions from the stainless casing, springs, and current collecting plates. Thus, the model can also partially reflect the conditions in pouch cells. The total areal mass of the cell (*m_cell_*, mg cm^−2^) includes the areal mass of all essential components to function, including a Zn negative electrode (*m_N_*), carbon positive electrode (*m_P_*), current collector (*m_cc_*), separator (*m_sp_*), and electrolyte (*m_E_*) (Equation 8).

(8) $$m_{\mathrm{cell}}=m_{N}+m_{P}+m_{\mathrm{cc}}+m_{\mathrm{sp}}+m_{E}$$

We consider that Zn foil serves as both an active material and a current collector in negative electrodes; thus, *m_N_* can be computed with the areal capacity (*Q*_*A*,*P*_) of positive electrodes and the gravimetric specific capacity of Zn metal (*Q*_*g*,*Zn*_ of 820 mAh g^−1^). Further, if the N/P ratio, the areal mass loading of active carbon materials (*m_c_*), and the gravimetric specific capacity of active carbon materials (*Q*_*g*,*c*_) are known, then *m_N_* can be directly correlated with the areal mass loading of carbon materials (*m_c_*) in the positive electrode (Equation 9).

(9) $$m_{N}=m_{Zn}=\;\;\frac{\frac{N}{P}\times Q_{A,P}\;}{10^{-3}\times Q_{g,Zn}}=\frac{\frac{N}{P}\times Q_{g,c}\times m_{c}\;}{Q_{g,Zn}}\;$$

Positive electrodes are composed of carbon materials (*m_c_*) as active materials for energy storage, conductive additives (*m_ca_*) (e.g., carbon black), and binders (*m_B_*) (e.g., polyvinylidene fluoride (PVDF)).^[^ ^18^ ^]^ We set their mass ratio (*m_c_*: *m_ca_*:*m_B_*) as 8:1:1 in the device model, similar to the mass ratio used in many recent studies. We vary *m_c_* (as *x*) from 2 to 50 mg cm^−2^ to benchmark with the mass loading of active carbon materials used in commercial supercapacitors.^[^ ^7a^ ^]^ Correspondingly, the areal mass of positive electrodes (*m_P_*) can be expressed as (Equation 10):

(10) $$\;m_{P}=m_{c}+m_{ca}+m_{B}=1.25\;m_{c}$$

Considering using a Ti foil (10 µm thickness) as the current collector, the areal mass of the current collector (*m_cc_*) would be 4.42 mg cm^−2^ (as experimentally measured). Suppose a standard separator in aqueous supercapacitors (Celgard 3501‐PP hydrophilic surfactant‐coated monolayer microporous membrane) is used. In that case, it has a porosity (*ε*) of 55%, an areal density (*ρ_sp_*) of 14 g m^−2^, and a thickness (*t*) of 25 µm. The areal mass of the separator (*m_sp_*) would be 1.4 g cm^−2^.^[^ ^19^ ^]^

2 m ZnSO_4_ aqueous solution is used as the electrolyte in the model due to its high ionic conductivity (≈58.7 mS cm^−1^) and broad adoption in many recent studies.^[^ ^4c^ ^]^ Two scenarios of electrolyte usage are considered: lean electrolyte condition and flooding electrolyte condition. One scenario is the ideal lean electrolyte condition where a minimum electrolyte (*m*_*E*,*min*_, mg cm^−2^) is just enough to fill pores in active carbon materials (i.e., YP‐50F) (*m*_*E*,*c*_, mg cm^−2^), conductive additives (i.e., carbon black) (*m*_*E*,*ca*_, mg cm^−2^), and the separator (i.e., Celgard 3501‐PP) (*m*_*E*,*sp*_, mg cm^−2^) (Equation 11).^[^ ^15^, ^20^ ^]^

(11) $$m_{E,min}=m_{E,c}+m_{E,ca}+m_{E,sp}$$

*m*_*E*,*c*_and *m*_*E*,*ca*_can be estimated by the density of electrolyte (ρ_*E*_) (e.g., 1.31 g cm^−3^ for 2 m ZnSO_4_ aqueous solution at 25 °C), the areal mass (*m_c_*) and specific pore volume (*V*_*pore*,*c*_) of active carbon materials (e.g., YP‐50F with *V*_*pore*,*c*_ of 0.74 cm^3^ g^−1^), and the areal mass (*m_ca_*) and specific pore volume (*V*_*pore*,*ca*_) of conductive additives (e.g., Super‐P with *V*_*pore*,*ca*_ of 0.2429 cm^3^ g^−1^) (Equations 12 and 13).

(12) $$m_{E,c}=m_{c}\;\times V_{pore,c}\times \rho_{E}\;$$

(13) $$m_{E,ca}=m_{ca}\;\times V_{pore,ca}\times \rho_{E}$$

The electrolyte in the separator (*m*_*E*,*sp*_, mg cm^−2^) can be estimated from the separator thickness (*t*) and its porosity (*ε*) in Equation (14):

(14) $$m_{E,sp}=t\times \varepsilon \times \rho_{E}\;$$

When the mass loading of active carbon materials increases, more electrolytes are required to fill up pores in the positive electrode. As shown in Table S3 (Supporting Information), when *m_c_* increases from 2 to 50 mg cm^−2^, the E/C ratio varies from 1.91 to 1.05 in a relatively narrow range. Hence, we set the model's E/C ratio at 2 as the threshold for the lean electrolyte condition.

The other scenario is the flooding electrolyte condition when the electrolyte is oversupplied. *m_E_* can be quantified by the E/C ratio and *m_c_* (Equation 7).^[^ ^16b^ ^]^

Combining all cell components discussed above, *m_cell_* can be re‐written as a function of *m_c_* (*x*), where N/P ratio, *Q*_*g*,*c*_, and E/C can take a range of different values (see the Table S4, Supporting Information):

(15) $$m_{cell}=\frac{\frac{N}{P}\times Q_{g,c}\times m_{c}\;}{Q_{g,Zn}}+1.25\;m_{c}+5.82+\frac{E}{C}\times m_{c}$$

A hybrid capacitor's negative and positive electrodes can be considered two capacitors connected in series.^[^ ^10^ ^]^ We assume the Zn negative and activated carbon positive electrodes have the same geometric surface area. The areal capacity of the Zn negative electrode is much larger than that of the carbon positive electrode (*Q*_*A*,*N*_ ≫ *Q*_*A*,*P*_). Thus, the areal capacity of the cell (*Q*_*A*,*cell*_, mAh cm^−2^) can be approximated by *Q*_*A*,*P*_ (Equation 16).^[^ ^3^, ^4^ ^]^

(16) $$Q_{A,cell}=\frac{1}{\frac{1}{Q_{A,N}}+\frac{1}{Q_{A,P}}}\;\approx Q_{A,P}$$

The model cell's energy density (*E*_*g*,*cell*_, Wh kg^−1^) can be calculated using the voltage window (*U*, 0.2–1.8 V), *Q*_*A*,*cell*_ and *m_cell_* (Equation 17).

(17) $$E_{g,cell}=\frac{U\times Q_{A,cell}}{10^{-3}\times m_{cell}}$$

*m_c_* is varied from 2 to 50 mg cm^−2^ to cover various potential electrode conditions. *Q*_*g*,*c*_ of commercial activated carbon at 50 mAh g^−1^ is adopted as the baseline for the specific capacity of active carbon materials,^[^ ^21^ ^]^ and it is then incrementally raised to 200 mAh g^−1^ to cover various high‐capacity carbon materials.^[^ ^4^, ^22^ ^]^

The E/C ratio changes from 2 for the lean electrolyte condition to 50 for the flooding electrolyte condition. The N/P ratio also increases from 2, with one‐time excessive metallic Zn, corresponding to a high 50% Zn utilization condition, to 300, with 299 times excessive metallic Zn, corresponding to a low 0.33% Zn utilization condition.

## 4 Influences of the Four Critical Parameters

### 4.1 The Influences of *m_c_* and *Q*_*g*,*c*_

**Figure 2:**
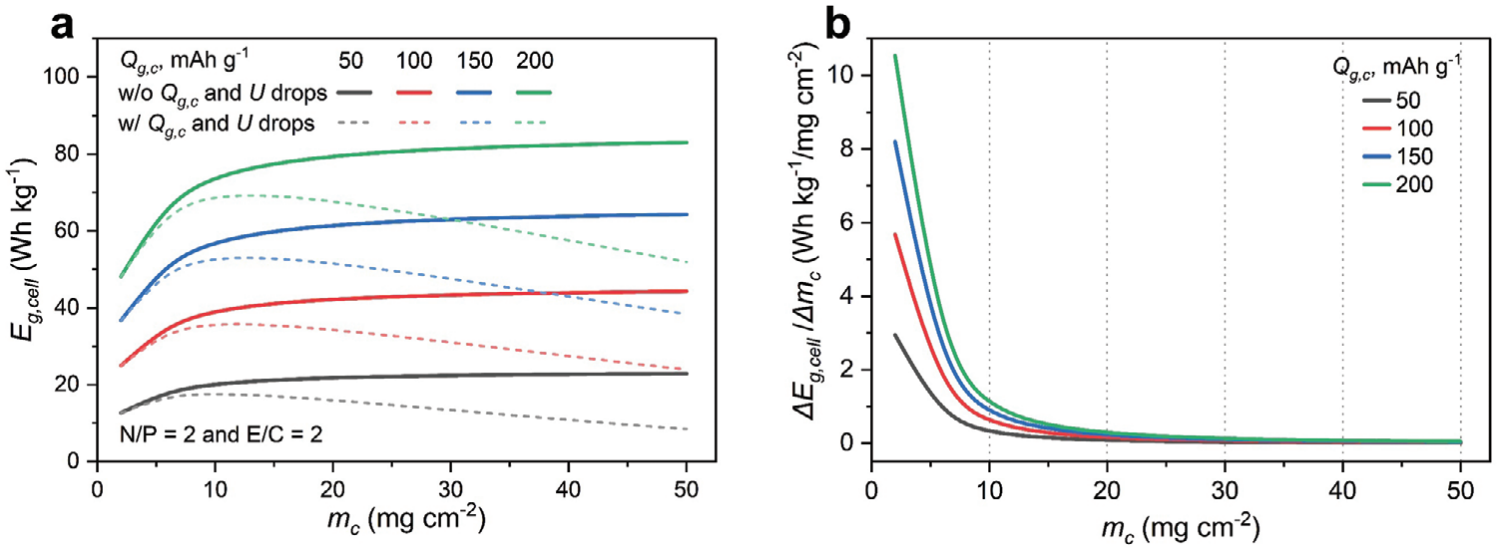
a) Cell gravimetric energy density (*E*_*g*,*cell*_) at different areal mass loadings of active carbon materials (*m_c_* from 2 to 50 mg cm^−2^) and carbon‐specific capacities (*Q*_*g*,*c*_ from 50 to 200 mAh g^−1^) in carbon positive electrodes with fixed N/P (2) and E/C (2) ratios. Solid lines represent ideal energy storage conditions that *Q*_*g*,*c*_ and *U* do not change with the increase of *m_c_*. Dash lines represent relatively more realistic conditions that *Q*_*g*,*c*_ and *U* drop with the increase of *m_c_*. b) The increasing rate of *E*_*g*,*cell*_ at different *m_c_* and *Q*_*g*,*c*_.

We first fix the N/P and E/C ratios at 2 with the lean electrolyte and high Zn utilization conditions and then examine how *E*_*g*,*cell*_ changes at different *m_c_* from 2 to 50 mg cm^−2^ and *Q*_*g*,*c*_ from 50 to 200 mAh g^−1^. **Figure** **2**a shows that *E*_*g*,*cell*_ increases with raising *m_c_* at all *Q*_*g*,*c*_. For example, at *Q*_*g*,*c*_ of 200 mAh g^−1^, *E*_*g*,*cell*_ climbs sharply from 48.1 to 74.1 Wh kg^−1^ as *m_c_* increases from 2 to 10 mg cm^−2^. The elevation in *E*_*g*,*cell*_ mainly results from more active carbon materials in positive electrodes, improving their areal capacity. Nevertheless, the impact of *m_c_* diminishes as it approaches higher values. Beyond 10 mg cm^−2^, *E*_*g*,*cell*_ enters a plateau region, with only negligible increases. For example, at *Q*_*g*,*c*_ of 150 mAh g^−1^, *E*_*g*,*cell*_ experiences a considerable boost from 36.7 Wh kg^−1^ at 2 mg cm^−2^ to 57.2 Wh kg^−1^ at 10 mg cm^−2^. In contrast, beyond 10 mg cm^−2^, only a marginal rise of ≈7.0 Wh kg^−1^ when *m_c_* increases from 10 to 50 mg^−1^. This trend becomes more evident when examining the increasing rate of *E*_*g*,*cell*_ (Δ*E*_*g*,*cell*_/Δ*m_c_*, Wh kg^−1^/mg cm^−2^) (Supporting Information). Figure 2b shows that Δ*E*_*g*,*cell*_/Δ*m_c_* undergoes an exponential decline as *m_c_* builds up at all *Q*_*g*,*c*_ values. Specifically, if *m_c_* exceeds 10 mg cm^−2^, Δ*E*_*g*,*cell*_/Δ*m_c_* becomes insignificant. This suggests the presence of a critical *m_c_* to deliver the optimum *E*_*g*,*cell*_. Beyond this threshold, adding more active carbon materials into carbon positive electrodes does not yield further improvements in *E*_*g*,*cell*_.

Further, the above calculations are based on an ideal energy storage condition without considering increased mass transfer and electrical contact resistances in thick positive electrodes. In practical scenarios, increasing *m_c_* would inevitably cause drops in the device's potential window and *Q*_*g*,*c*_ of active carbon materials. Based on several recent experimental studies, we can consider a ≈0.5 mAh g^−1^ drop in *Q*_*g*,*c*_,^[^ ^21^ ^]^ and ≈10 mV drop in *U* per 1 mg cm^−2^ increase in *m_c_*. ^[^ ^23^ ^]^ Dash lines in Figure 2a show the changes of *E*_*g*,*cell*_ under this more realistic energy storage condition. Thus, the optimal *E*_*g*,*cell*_ could be achieved when *m_c_* is ≈10 mg cm^−2^ at 17.7, 35.9 Wh kg^−1^ for *Q*_*g*,*c*_ of 50, 100 mAh g^−1^ and 14 mg cm^−2^ at 53.0, 69.1 Wh kg^−1^ for *Q*_*g*,*c*_ of 150, 200 mAh g^−1^, respectively. *E*_*g*,*cell*_ gradually falls with the increase of *m_c_* beyond 10 mg cm^−2^. Compared to *m_c_*, *Q*_*g*,*c*_ has a more significant effect on *E*_*g*,*cell*_. *E*_*g*,*cell*_ increases by ≈20.0 Wh kg^−1^ with each incremental increase of *Q*_*g*,*c*_ in 50 mAh g^−1^ (Figure 2a). Figure 2b also shows that active carbon materials with a higher *Q*_*g*,*c*_ have a larger Δ*E*_*g*,*cell*_/Δ*m_c_* with the surge of *m_c_*. For instance, at *m_c_* of 2.0 mg cm^−2^, carbon materials with *Q*_*g*,*c*_ of 200 mAh g^−1^ increase by 10.5 Wh kg^−1^ / mg cm^−2^, which is much higher than that of carbon materials with *Q*_*g*,*c*_ of 50 mAh g^−1^ at 2.95 Wh kg^−1^ / mg cm^−2^. However, this difference disappears when *m_c_* is higher than 20 mg cm^−2^. Based on the above analysis, under low N/P and E/C ratios, an active carbon positive electrode with *m_c_* of 10 mg cm^−2^ and *Q*_*g*,*c*_ of 150 mAh g^−1^ would deliver a *E*_*g*,*cell*_ of 57.2 Wh kg^−1^. Such ZIHCs would be very competitive for energy storage applications.

### 4.2 The Influence of the N/P Ratio

**Figure 3:**
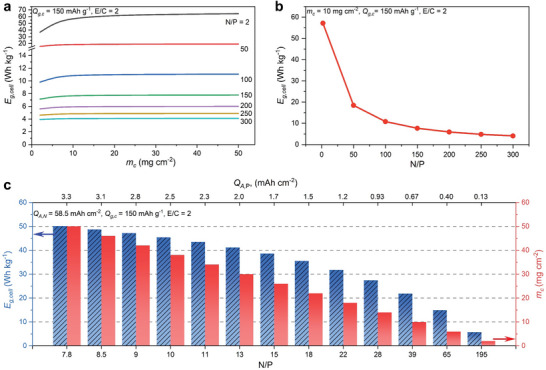
a) Cell gravimetric energy density (*E*_*g*,*cell*_) at different N/P ratios (from 2 to 300) and areal mass loadings of active carbon materials in the carbon positive electrode (*m_c_* from 2 to 50 mg cm^−2^) with fixed *Q*_*g*,*c*_ (150 mAh g^−1^) and E/C ratio (2). b) The change of *E*_*g*,*cell*_ at different N/P ratios with fixed *m_c_* (10 mg cm^−2^), *Q*_*g*,*c*_ (150 mAh g^−1^), and E/C ratio (2). c) *E*_*g*,*cell*_ (left) and the corresponding *m_c_* at different N/P ratios (bottom) and *Q*_*A*,*P*_ (top) based on a Zn foil with a thickness of 100 µm (*Q*_*A*,*N*_ of 58.5 mAh cm^−2^), *Q*_*g*,*c*_ of 150 mAh g^−1^, and E/C ratio (2).

Next, we examine how the N/P ratio would affect *E*_*g*,*cell*_. We fix the E/C ratio at 2 (lean electrolyte condition) and *Q*_*g*,*c*_ at 150 mAh g^−1^ (a high‐capacity active carbon material), and vary the N/P ratio from 2 to 300 and *m_c_* from 2.0 to 50 mg cm^−2^. **Figure** **3**a shows that *E*_*g*,*cell*_ enters a plateau when *m_c_* is larger than 10 mg cm^−2^ at all N/P ratios. *E*_*g*,*cell*_ descends significantly with the increase of the N/P ratio from 2 to 300 for all *m_c_*. Specifically, *E*_*g*,*cell*_ at the N/P of 2 (DOD, 50%) reaches its plateau at 62.0 Wh kg^−1^, ≈15 times larger than *E*_*g*,*cell*_ of 4.0 Wh kg^−1^ at the N/P of 300. The changes of *E*_*g*,*cell*_ show a nonlinear correlation with the changes in N/P ratios. As illustrated in Figure 3b, when *m_c_* is fixed at 10 mg cm^−2^, *E*_*g*,*cell*_ drops quickly from 57.2 to 10.9 Wh kg^−1^ when the N/P ratio increases from 2 to 100, and has minor changes when the N/P ratio further increases to 300. These results indicate that excessive Zn negative electrodes are detrimental to achieving a high *E*_*g*,*cell*_. More usefully, the results imply that lowering the N/P ratio from 50 to 2 would more likely result in the improvement of *E*_*g*,*cell*_.

In practical ZIHC fabrication, achieving a small N/P ratio using ultrathin Zn foils (e.g., less than 10 µm in thickness) is challenging due to their mechanical fragileness and instability upon multiple charge/discharge cycles. On the other hand, the N/P ratios can be modulated to some extent by varying the properties of carbon‐positive electrodes, specifically their *m_c_* and *Q*_*g*,*c*_. Figure 3c shows the relationship among *m_c_*, N/P ratio and *E*_*g*,*cell*_ when we fix the thickness of Zn foils at 100 µm and *Q*_*g*,*c*_ at 150 mAh g^−1^. 100 µm thickness is considered in our model because Zn foils also serve as current collectors at negative electrodes. If their thickness is too thin, additional current collectors would be required. The maximum *E*_*g*,*cell*_ of 50.1 Wh kg^−1^ would be possible at the N/P ratio of 7.8 and *m_c_* of 50 mg cm^−2^. In contrast, the lowest *E*_*g*,*cell*_ of 5.74 Wh kg^−1^ would have an N/P ratio of 195 and *m_c_* of 2.0 mg cm^−2^. To meet a benchmark *E*_*g*,*cell*_ of 30 Wh kg^−1^ (comparable to lead acid batteries), a minimum N/P ratio of 22 is required with *m_c_* of 18 mg cm^−2^ to deliver 31.8 Wh kg^−1^. Further, considering the early discussion about the optimum window of *m_c_* between 10 and 20 mg cm^−2^, the desirable N/P ratio in ZIHCs with 100 µm thickness Zn foil negative electrodes should be ≈20.

### 4.3 The Influence of the E/C Ratio

**Figure 4:**
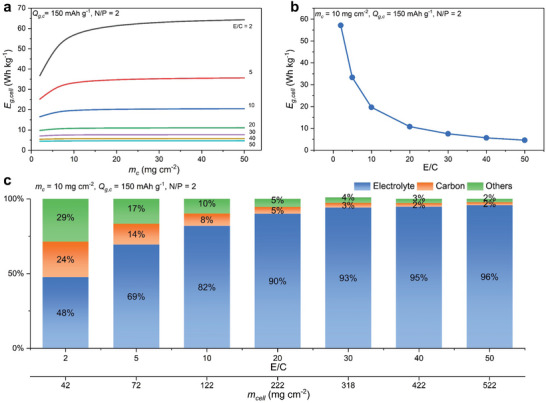
a) Cell gravimetric energy density (*E*_*g*,*cell*_) at different E/C ratios (from 2 to 50) and areal mass loadings of active carbon materials in the carbon positive electrode (*m_c_* from 2 to 50 mg cm^−2^) with fixed *Q*_*g*,*c*_ (150 mAh g^−1^) and N/P ratio (2). b) The change of *E*_*g*,*cell*_ at different E/C ratios with fixed *m_c_* (10 mg cm^−2^), *Q*_*g*,*c*_ (150 mAh g^−1^) and N/P ratio (2). c) The mass distribution of key cell components, including electrolyte, active carbon materials, and others (i.e., Zn foil, conductive additive, binder, separator, and current collector) with a fixed *m_c_* (10 mg cm^−2^), *Q*_*g*,*c*_ (150 mAh g^−1^) and N/P ratio (2).

Last, we analyze the effect of the E/C ratio on *E*_*g*,*cell*_. **Figure** **4**a shows that similar to the N/P ratio shown in Figure 3a, *E*_*g*,*cell*_ enters a plateau when *m_c_* is larger than 10 mg cm^−2^ at all E/C ratios. A high E/C ratio (e.g., 50 of a flooded electrolyte condition) would result in a low *E*_*g*,*cell*_ of ≈4.0 Wh kg^−1^. In contrast, a low E/C ratio (e.g., at 2 of a lean electrolyte condition) can deliver a high *E*_*g*,*cell*_ of ≈62 Wh kg^−1^. The changes of *E*_*g*,*cell*_ also show a nonlinear correlation with the changes in E/C ratios. Figure 4b shows that *E*_*g*,*cell*_ decreases quickly from 57.2 to 4.6 Wh kg^−1^ at a fixed *m_c_* of 10 mg cm^−2^ when the E/C ratio increases from 2 to 10 and has a much smaller decline when the E/C ratio further increases to 50.

To better illustrate the role of electrolytes among all cell components, we calculate the mass distribution of major cell components, including electrolytes, active carbon materials, and others (i.e., Zn foil, conductive additive, binder, separator, and current collector) for the model ZIHC with fixed *m_c_* (10 mg cm^−2^), *Q_g,c_* (150 mAh g^−1^) and N/P ratio (2). Figure 4c displays that the mass of electrolytes contributes to almost half of the whole cell (48%), even at the lean electrolyte condition (E/C of 2), surpassing all other cell components (i.e., 29% for all others and 24% for active carbon materials). When the E/C ratio is at 50, the mass proportion of electrolytes is 96%, while the proportion of active carbon materials drops to merely 2%. It would be essential to keep the E/C ratio below 5 if the desired *E*_*g*,*cell*_ is above 30 Wh kg^−1^.

## 5 Conclusion

Our analysis shows that increasing *m_c_* to boost *E*_*g*,*cell*_ would hit a plateau when *m_c_* is higher than 10 mg cm^−2^. Considering drops in the device's potential window and *Q*_*g*,*c*_ at higher *m_c_*, the optimal *m_c_* should be kept below 20 mg cm^−2^. Thus, we recommend having *m_c_* in the range from 10 to 20 mg cm^−2^ to obtain optimal *E*_*g*,*cell*_. Achieving such high *m_c_* faces several problems. An electrode with a thicker active carbon material layer would increase mass transfer distance with higher tortuosity and raise contact resistance among carbon particles, resulting in inefficient ion and electron transfers.^[^ ^9^, ^24^ ^]^ Further, a thicker active carbon material layer may also have poor mechanical integrity and adhesion to the current collector, resulting in carbon layer delamination. These effects would trigger the decay in *Q*_*g*,*c*_, especially under fast charge/discharge rates.^[^ ^13^, ^25^ ^]^ Recent studies have adopted different approaches to address these problems. For example, incorporating hierarchical pore channels and controllable porosity into a thick active carbon material layer can improve the mass transfer of ions, which has been attempted by 3D printing,^[^ ^26^ ^]^ salt‐based templating,^[^ ^11c^ ^]^ and layer‐to‐layer spray coating.^[^ ^27^ ^]^ Mechanically robust and electrically conductive materials, such as carbon nanotubes^[^ ^28^ ^]^ and carbon fibers,^[^ ^29^ ^]^ have been incorporated into a thick active carbon material layer to create an electrically conductive network to assist electron transfer and improve carbon layers’ mechanical stability.

*Q*_*g*,*c*_of active carbon materials plays a fundamental role in governing *E*_*g*,*cell*_. It is desirable to use active carbon materials with *Q*_*g*,*c*_ of at least 100 mAh g^−1^ so that the resulting ZIHCs would have the potential to deliver *E*_*g*,*cell*_ above 30 Wh kg^−1^. In general, conductive carbon materials with a large specific surface area are required to deliver a high *Q*_*g*,*c*_. A recent study also identified the critical role of structure disorder.^[^ ^30^ ^]^ Further, pores with a size close to that of solvated Zn ions can also facilitate more rapid adsorption and desorption with low binding energy.^[^ ^31^ ^]^ Doping heteroatoms into carbon materials has often been used to boost their *Q*_*g*,*c*_ via additional pseudocapacitance; however, carbon materials doped with a high concentration of heteroatoms may suffer from poor long‐term stability.^[^ ^31a^ ^]^

A high N/P ratio is detrimental to *E*_*g*,*cell*_. Our analysis shows that the N/P ratio should be controlled below 20. However, the adverse effects of Zn corrosion and dendrite growth would aggravate at a low N/P ratio, resulting in a shortened cycle life.^[^ ^14a^ ^]^ Various approaches, ranging from Zn surface functionalization, coating Zn surface with organic or inorganic materials,^[^ ^32^ ^]^ to 3D structuring of Zn foil,^[^ ^33^ ^]^ have been attempted in recent studies to improve the performance of Zn electrodes. It is essential to objectively evaluate the effectiveness of these Zn electrode modification methods at conditions relevant to practical applications, i.e., with proper values for the four parameters discussed above.

Similarly, a low E/C ratio is desirable for obtaining a high *E*_*g*,*cell*_. A low E/C ratio below 5 would be desirable in practical ZIHCs. Unfortunately, harmful efforts of electrolyte‐related parasitic reactions, such as hydrogen evolution reactions, are vastly amplified at low E/C ratios. For example, a rapid electrolyte dry‐up may occur, resulting in poor cycling stability and fast cell performance fading.^[^ ^34^ ^]^ Further, inadequate electrolytes would have difficulty infiltrating thick carbon electrodes, resulting in insufficient wetting. These would increase cell internal resistance and undermine ion transfer.^[^ ^16^, ^35^ ^]^ Recent studies have also explored quasi‐solid‐state electrolytes, such as hydrogels, to reduce the amounts of electrolytes and eliminate the use of separators. The interactions between functional groups on hydrogels and Zn surface may also inhibit Zn dendrite formation.^[^ ^36^ ^]^ Nonetheless, the total mass of hydrogel electrolytes used in ZIHCs needs to be controlled at a low E/C ratio to deliver a high *E*_*g*,*cell*_.

It should be noted that the emphasis on the four essential parameters and their influences on ZIHCs’ device energy density does not intend to discourage fundamental research studies focusing on improving various components of ZIHCs, such as carbon electrode materials, Zn electrodes, and electrolytes. Studying their intrinsic electrochemical properties under simplified or idealized conditions has undoubtedly provided valuable findings to drive this research field forward. However, we should be cautious when predicting practical device performance based on results obtained from these simplified or idealized testing conditions. As shown in our analyses, such predictions are likely to be unreliable.

In conclusion, *E*_*g*,*cell*_ of ZIHCs depends on four key parameters: *m_c_*, *Q*_*g*,*c*_, N/P and E/C ratios. A high *m_c_* (10–20 mg cm^−2^), high *Q*_*g*,*c*_ (>100 mAh g^−1^), a low N/P ratio (<20) and a low E/C ratio (<5) are required simultaneously to deliver ZIHCs with energy density relevant to many potential practical applications (e.g., >30 Wh kg^−1^). Notably, the device performance results obtained by improving one parameter without considering the other three are often unreliable. We hope this work can inspire more future studies to be conducted holistically to realize realistic applications of ZIHCs.

## Conflict of Interest

The authors declare no conflict of interest.

## Supporting information



Supporting Information

## References

[1] a) Z. X. Zhu , T. L. Jiang , M. Ali , Y. H. Meng , Y. Jin , Y. Cui , W. Chen , Chem. Rev. 2022, 122, 16610; 36150378 10.1021/acs.chemrev.2c00289

[2] a) E. Frackowiak , F. Béguin , Carbon 2001, 39, 937;

[3] a) Y. Shao , M. F. El‐Kady , J. Sun , Y. Li , Q. Zhang , M. Zhu , H. Wang , B. Dunn , R. B. Kaner , Chem. Rev. 2018, 118, 9233; 30204424 10.1021/acs.chemrev.8b00252

[4] a) J. Yin , W. Zhang , N. A. Alhebshi , N. Salah , H. N. Alshareef , Adv. Energy Mater. 2021, 11, 2100201; 10.1021/acsami.1c06543 34047542

[5] Y. Gogotsi , P. Simon , Science 2011, 334, 917. 22096182 10.1126/science.1213003

[6] a) S. S. Shinde , N. K. Wagh , C. H. Lee , D. H. Kim , S. H. Kim , H. D. Um , S. U. Lee , J. H. Lee , Adv. Mater. 2023, 35, 2303509; 10.1002/adma.202303509 37752717

[7] a) L. Chang , Y. H. Hu , Matter 2019, 1, 596;

[8] a) C. Li , S. Jin , L. A. Archer , L. F. Nazar , Joule 2022, 6, 1733;

[9] Y. Kuang , C. Chen , D. Kirsch , L. Hu , Adv. Energy Mater. 2019, 9, 1901457.

[10] a) J. W. Gittins , Y. Chen , S. Arnold , V. Augustyn , A. Balducci , T. Brousse , E. Frackowiak , P. Gómez‐Romero , A. Kanwade , L. Köps , P. K. Jha , D. Lyu , M. Meo , D. Pandey , L. Pang , V. Presser , M. Rapisarda , D. Rueda‐García , S. Saeed , P. M. Shirage , A. Ślesiński , F. Soavi , J. Thomas , M.‐M. Titirici , H. Wang , Z. Xu , A. Yu , M. Zhang , A. C. Forse , J. Power Sources 2023, 585, 233637;

[11] a) Y. Lu , Z. Li , Z. Bai , H. Mi , C. Ji , H. Pang , C. Yu , J. Qiu , Nano Energy 2019, 66, 104132;

[12] F. Wei , Y. Zeng , Y. Guo , J. Li , S. Zhu , S. Gao , H. Zhang , X. He , Chem. Eng. J. 2023, 468, 143576.

[13] S.‐H. Park , P. J. King , R. Tian , C. S. Boland , J. Coelho , C. Zhang , P. McBean , N. McEvoy , M. P. Kremer , D. Daly , J. N. Coleman , V. Nicolosi , Nat. Energy 2019, 4, 560.

[14] a) B. Sambandam , V. Mathew , F. Ahmad Nurul , S. Kim , M. Song , J. Kim , ACS Energy Lett. 2024, 9, 3058;

[15] a) H. Wang , W. Ye , Y. Yang , Y. Zhong , Y. Hu , Nano Energy 2021, 85, 105942;

[16] a) G. Zampardi , F. L. Mantia , Nat. Commun. 2022, 13, 687; 35115524 10.1038/s41467-022-28381-x PMC8814157

[17] S. Chen , C. Niu , H. Lee , Q. Li , L. Yu , W. Xu , J.‐G. Zhang , E. J. Dufek , M. S. Whittingham , S. Meng , J. Xiao , J. Liu , Joule 2019, 3, 1094.

[18] C. Wang , Z. X. Pei , Q. Q. Meng , C. M. Zhang , X. Sui , Z. W. Yuan , S. J. Wang , Y. Chen , Angew. Chem., Int. Ed. 2021, 60, 990. 10.1002/anie.202012030 32969140

[19] Y. Zhang , X. Li , L. Fan , Y. Shuai , N. Zhang , Cell Rep. Phys. Sci. 2022, 3, 100824.

[20] J. Wu , X. Zhang , Z. Ju , L. Wang , Z. Hui , K. Mayilvahanan , K. J. Takeuchi , A. C. Marschilok , A. C. West , E. S. Takeuchi , G. Yu , Adv. Mater. 2021, 33, 2101275. 10.1002/adma.202101275 34028911

[21] Z. Li , D. Chen , Y. An , C. Chen , L. Wu , Z. Chen , Y. Sun , X. Zhang , Energy Storage Mater. 2020, 28, 307.

[22] M. Wu , X. Hu , W. Zheng , L. Chen , Q. Zhang , Chem. Eng. J. 2023, 466, 143077.

[23] a) N. Guo , W. Luo , R. Guo , D. Qiu , Z. Zhao , L. Wang , D. Jia , J. Guo , J. Alloys Compd. 2020, 834, 155115;

[24] a) J. Wu , Z. Ju , X. Zhang , K. J. Takeuchi , A. C. Marschilok , E. S. Takeuchi , G. Yu , Nano Lett. 2021, 21, 9339; 34669404 10.1021/acs.nanolett.1c03724

[25] Y. Chen , B. Zhao , Y. Yang , A. Cao , Adv. Energy Mater. 2022, 12, 2201834.

[26] Y. Liu , S. Zheng , J. Ma , X. Wang , L. Zhang , P. Das , K. Wang , Z. S. Wu , Adv. Energy Mater. 2022, 12, 2200341.

[27] S. H. Lee , C. Johnston , P. S. Grant , ACS Appl. Mater. Interfaces 2019, 11, 37859. 31553158 10.1021/acsami.9b14478

[28] Z. Xu , Z. Sun , J. Shan , S. Jin , J. Cui , Z. Deng , M. H. Seo , X. Wang , Adv. Funct. Mater. 2024, 34, 2302818.

[29] C. Wan , J. Huang , K. Chen , C. Jiang , Q. Wu , P. Huang , Q. Xu , S. Qin , H. Xie , Energy Storage Mater. 2024, 69, 103384.

[30] X. Liu , D. Lyu , C. Merlet , M. J. A. Leesmith , X. Hua , Z. Xu , C. P. Grey , A. C. Forse , Science 2024, 384, 321. 38635707 10.1126/science.adn6242

[31] a) W. Jian , W. Zhang , X. Wei , B. Wu , W. Liang , Y. Wu , J. Yin , K. Lu , Y. Chen , H. N. Alshareef , X. Qiu , Adv. Funct. Mater. 2022, 32, 2209914;

[32] C. Nie , G. Wang , D. Wang , M. Wang , X. Gao , Z. Bai , N. Wang , J. Yang , Z. Xing , S. Dou , Adv. Energy Mater. 2023, 13, 2300606.

[33] L. Li , Y. Zheng , J. Xu , B. Peng , G. Zhu , J. Wu , L. Ma , Z. Jin , ACS Energy Lett. 2024, 9, 3269.

[34] Y. Xu , X. Zhou , Z. Chen , Y. Hou , Y. You , J. Lu , Mater. Today 2023, 66, 339.

[35] H. Kwon , J. Baek , H.‐T. Kim , Energy Storage Mater. 2023, 55, 708.

[36] a) Y. Wang , Q. Li , H. Hong , S. Yang , R. Zhang , X. Wang , X. Jin , B. Xiong , S. Bai , C. Zhi , Nat. Commun. 2023, 14, 3890; 37393327 10.1038/s41467-023-39634-8 PMC10314915

