# Supplementary material for: Zinc Ion Hybrid Capacitors: Four Essential Parameters Determining Device Energy Density
Source: Adv Sci (Weinh). 2024 Nov 6;11(48):2408997. doi: 10.1002/advs.202408997 (PMC11672303; doi:10.1002/advs.202408997)
Supplement: Supplementary file 1 — Supporting Information [file ADVS-11-2408997-s001.docx]

**Supporting Information**

Zinc ion hybrid capacitors: Four essential parameters determining device energy density

Jiacheng Wu, Di Zhu, Yuqi Pan, Justin Prabowo, Li Wei, Yuan Chen*

School of Chemical and Biomolecular Engineering, The University of Sydney, Darlington, NSW South Wales, 2006, Australia

Corresponding Author

* [yuan.chen@sydney.edu.au](mailto:yuan.chen@sydney.edu.au) (Y.C.)

**Table S1** A summary of the structure and performance of recently reported zinc ion hybrid capacitors with different carbon cathodes.

| **Negative electrode materials** | **Zn foil thickness (*μ*m)** | **Positive electrode materials** | **Current collector of positive electrodes** | **Carbon**  **material**  **mass loading (mg cm^-2^)** | **Calculated N/P ratio/DOD** | **Electrolytes** | **Electrolyte**  **amount (*μ*L)** | **Separators** | **Voltage (V)** | **Reported specific capacity (mAh g^-1^)/**  **Current density (A g^-1^)** | **Reported**  **peak**  **energy density**  **(Wh kg^-1^)** | **Reported**  **peak**  **power density**  **(kW kg^-1^)** | **Year** | **Refs.** |
| --- | --- | --- | --- | --- | --- | --- | --- | --- | --- | --- | --- | --- | --- | --- |
| Zn foil | - | Layered N/B co-doped carbon (LDC) | Graphite paper | 2 | - | 1 M ZnSO_4_ | - | Whatman filter paper separator | 0.2-1.8 | 127.7/0.5 | 97.6 | - | 2019 | ^[1]^ |
| Porous carbon  coated Zn | - | Porous carbon (PC-800) | Stainless steel mesh | 1.4-2.0 | - | 3 M Zn(ClO_4_)_2_ | - | Wood pulp/polyester fabric | 0-1.9 | 179.8 (340.7 F g^-1^)/0.1 | 104.8 | 48.8 | 2020 | ^[2]^ |
| Zn foil | 50 | Hierarchical porous activated carbon (HPAC) | Titanium foil | 0.6 | 211/0.47% | 3 M Zn(CF_3_SO_3_)_3_ | 200 | Glass fiber separator | 0-1.8 | 231.4/0.5 | ~100***** | 11.4 | 2020 | ^[3]^ |
| Zn foil | - | Pyrrolic-dominated N-doped pitch-derived carbon(NPC) | Carbon paper | 1.5 | - | 1 M ZnSO_4_ | - | - | 0.2-1.8 | 136.2/0.3 | 81.1 | 12.8 | 2021 | ^[4]^ |
| Zn foil | - | Amino-functionalized porous carbons (APC) | Carbon paper | 0.5-0.7 | - | 1M ZnCI_2_ | - | - | 0-2.0 | 250/0.3 | 130.9 | ~7.0 | 2022 | ^[5]^ |
| Zn foil | 50 | Bamboo-derived porous carbon (PBC-A900) | Stainless steel mesh | 5 | 41/2.4% | 1 M Zn(CF_3_SO_3_)_2_ | - | Glass fiber separator (Whatman) | 0.2-1.8 | 142.8 (321.3F g^-1^)/1.0 | 114.2 | ~10***** | 2022 | ^[6]^ |
| Zn foil | 250 | N and S-doped in porous carbon dodecahedra (N, S-PCD) | Carbon paper | 0.9 | 1218/0.082% | 2 M ZnSO_4_ | - | Whatman filter paper separator | 0.2-1.8 | 133.4/0.2 | 106.7 | 16***** | 2022 | ^[7]^ |
| Zn foil | - | Oxygen-rich porous carbons (ORC-900) | Stainless steel mesh | 2.0-3.0 | - | 1 M ZnSO_4_ | - | Whatman glass microfibers | 0.2-1.8 | 136.9 (308F g^-1^)/0.5 | 136.5 | 12.5 | 2022 | ^[8]^ |
| Zn foil | - | Nitrogen-enriched mesoporous carbon nanosheets (NPCNs) | Carbon cloth | 1.1 | - | 2 M ZnSO_4_ | - | - | 0.15-1.7 | 210.7/0.5 | 198.2 | 5.466 | 2022 | ^[9]^ |
| Zn foil | - | N, P, and S co-doped 3D porous carbon (HPCS-900) | Titanium foil | 0.8-0.9 | - | 3 M ZnSO_4_ | - | Whatman glass fiber | 0.1-1.7 | 104.7/0.1 | 90.17 | 32.26 | 2022 | ^[10]^ |
| Zn foil | - | N doped porous carbon nanosheet (NPCN-750) | - | 2.4 | - | 1 M Zn(CF_3_SO_3_)_2_ | - | Paper film membrane | 0.15-1.8 | 204.7/0.1 | 143 | 16.6 | 2023 | ^[11]^ |
| Zn foil | - | N and O co-doped carbon micro-foams (CMF-800) | Stainless steel mesh | 1.5-2.0 | - | 1 M Zn (CF_3_SO_3_)_2_/DMF | - | Glass fiber (GF/F, Whatman) | 0.2-1.8 | 111.0 (249.7 F g^-1^)/0.5 | 87 | 14.65 | 2023 | ^[12]^ |
| Zn foil | - | ZIF-derived N, O, F and Zn doped carbon microspheres (AC-H_2_O) | Carbon paper | 1 | - | 2 M ZnSO_4_ | - | - | 0.2-1.8 | 171.9/0.5 | 141.3 | 13.3 | 2023 | ^[13]^ |
| Zn foil | 50 | Biomass-derived N/O co-doped 2D arcuate carbon nanosheet (2DPC90) | Flexible graphite paper | 1 | 147/0.68% | 2 M ZnSO_4_ | - | Whatman filter paper separator | 0.2-1.8 | 198.4/0.2 | 155.6 | 18.9 | 2023 | ^[14]^ |
| Zn foil | 30 | N-S Co-doping honeycomb-like carbon (NS-OPC) | Stainless steel foil | 1 | 137/0.73% | 1 M ZnSO_4_ | - | filter paper | 0.01-1.8 | 128.1/0.2 | 71.2 | 6.064 | 2023 | ^[15]^ |
| Zn foil | - | Coralloidal B/N dual-doped carbon nano-bundles (BNC) | Carbon paper | 1 | - | 2 M ZnSO_4_ | - | Whatman glass microfiber filter | 0.05-1.8 | 204/0.2 | 178.7 | 17.5 | 2023 | ^[16]^ |
| Zn foil | - | N/O co-doped hydrophilic hierarchical porous carbon | Flexible graphite paper | 1 | - | 2 M ZnSO_4_ | - | Whatman filter paper | 0.2-1.8 | 225.3/0.1 | 201.4 | 83.6 | 2023 | ^[17]^ |
| Zn foil | - | Olive leaf-derived porous activated carbon (OLDC-750) | Steel mesh | 2 | - | 2 M ZnSO_4_ | - | Glass fiber separator | 0.2-1.8 | 136 (203.5 F g^-1^)/0.1 | 136.3 | 20 | 2023 | ^[18]^ |
| Zn foil | 500 | Glutinous rice-derived carbon (GRPC-A13) | Stainless steel foil | - | - | 2 M ZnSO_4_ | - | Glass microfiber filter (Whatman) | 0.2-1.8 | 177/0.5 | 116 | 8.0 | 2023 | ^[19]^ |
| Zn foil | - | MOF-derived Hierarchically porous carbon rods (MDPC-2) | Stainless steel mesh | - | - | 2 M Zn(CF_3_SO_3_)_2_ | - | Glass fiber (GF/D, Whatman) | 0-1.8 | 161.7 (323.4 F g^-1^)/0.5 | 145.5 | 45 | 2023 | ^[20]^ |
| Zn foil | 50 | N, S co-doped multi-adsorption sites porous carbon (HC-0.2) | Stainless steel sheet | 1 | 119/0.84% | 1 M ZnSO_4_ | - | Whatman glass fiber | 0.2-1.8 | 245.8/0.2 | 164.1 | 30.1 | 2024 | ^[21]^ |
| Zn foil | 80 | Oxygen-riched pitch-derived hierarchically porous carbon (HPC-1-4) | - | 1.6 | 142/0.7% | 1 M Zn(CF_3_SO_3_)_2_ | - | Glass fiber separator (Whatman) | 0.2-1.8 | 206.7/0.25 | 154.3 | 15.24 | 2024 | ^[22]^ |
| Zn foil | 100 | Pencil shavings derived porous carbon (PSC-A600) | Graphite paper | 2.0  24 | 159/0.63%  23.2/4.3% | 1 M Zn(CF_3_SO_3_)_2_ | - | Glass fiber separator (Whatman) | 0.2-1.8 | 183.7 (413.3 F g^-1^)/0.2  105 (236.3 F g^-1^)/0.2 | 147 | 15.7 | 2020 | ^[23]^ |
| Zn foil | 80 | Hierarchically porous nitrogen-doped carbon nanocage framework (PZC-A750) | Graphite paper | 2.0  15 | 189/0.53%  60/1.67% | 1 M Zn(CF_3_SO_3_)_2_ |  | Whatman glass fiber filter paper | 0.2-1.8 | 124/0.25  78/0.25 | 107.3  - | 16.6  - | 2021 | ^[24]^ |
| Zn foil | - | Rubidium-activated porous carbon (RbPC) | Stainless steel mesh | 1  30 | - | 1 M Zn(CF_3_SO_3_)_2_ | - | Glass fiber membrane | 0.2-1.8 | 260.4/0.5  103.6/0.1 | 178.2  - | 72.3  - | 2022 | ^[25]^ |
| Zn foil | - | N, O co-doped three-dimensional porous carbon networks (NPCNs) | Stainless steel mesh | 1.1  15.4 | - | 2 M ZnSO_4_ | - | Glass fiber membrane | 0.1-1.8 | 189.3/0.1  103.9/0.1 | 160.9  - | 17.0  - | 2023 | ^[26]^ |
| Zn foil | - | Spherical superstructures of N-doped carbon nanorods (SSNCR-800) | Stainless steel mesh | 1  50 | - | 1 M Zn(CF_3_SO_3_)_2_ | - | Glass fiber membrane | 0.2-1.8 | 131.6 (296 F g^-1^)/0.2  95.6 (215 F g^-1^)/0.2 | 181.2  115 | 31.1  - | 2023 | ^[27]^ |

*The reported peak energy density and power density are estimated from the relevant Ragone plot given in the work.

**A sample calculation of mass distribution in a coin cell structure:**

The areal mass of a 100 $\mu m$ thick Zn foil negative electrode ($m_{N}$) can be estimated by its density ($\rho$ = 7.14 mg cm^-2^) and thickness (t, *μ*m) as follows:

$m_{N}=m_{Zn}=\rho\times t=7.14\frac{g}{cm^{3}}\times1000\frac{mg}{g}\times100 \mu m\times{10}^{-4}\frac{cm}{\mu m}=71 mg cm^{-2}$ (S1)

The areal mass of a positive electrode ($m_{P}$) is calculated using the areal mass loading of activated carbon materials ($m_{c}$ = 2 mg cm^-2^) and in the mass ratio of 8 ($m_{c}$):1($m_{ca}$):1($m_{B}$):

$m_{P}=\frac{2\frac{mg}{cm^{2}}}{0.8}=2.5 mg cm^{-2}$ (S2)

The areal mass of 10 *μ*m Ti foil current collector ($m_{cc}$) is calculated by the experimental measurements:

$m_{cc}=4.42 mg cm^{-2}$ (S3)

The areal mass of a separator ($m_{sp}$) is calculated by converting the nominal basis weight of commercial GF/A Whatman separators (53 g m^-2^):

$m_{sp}=53\frac{g}{m^{2}}=\frac{53 g\times1000\frac{mg}{g}}{1 m^{2}\times\frac{10000 cm^{2}}{1 m^{2}}}=5.3 mg cm^{-2}$ (S4)

The electrolyte areal mass (*m_E_*) is calculated based on the density ($\rho_{E}$ = 1.31 g cm^-3^) and volume of 2 M ZnSO_4_ solution ($v$= 90 *μ*L) and divided by the electrode surface area (*A* = 1.13 cm^2^):

$m_{E}=\frac{\rho\times v}{A}=\frac{1.31\frac{g}{cm^{3}}\times1000\frac{mg}{g}\times90 \mu L\times{10}^{-3}\frac{mL}{\mu L}\times1\frac{cm^{3}}{mL}}{1.13 cm^{2}}=104 mg cm^{-2}$ (S5)

**Table S2.** Areal mass and mass distribution of all components in a representative ZIHC coin cell structure (the mass of cell casing and other supporting materials are excluded).

| **Cell components** | **Material details** | **Areal Mass**  **(mg cm^-2^)** | **Mass ratio (%)** |
| --- | --- | --- | --- |
| Negative electrode ($m_{N}$) | Zn foil ($m_{Zn}$) | 71 | 38 |
| Positive electrode ($m_{P}$) | Carbon materials ($m_{c}$), carbon black ($m_{ca}$), binder ($m_{B}$) | 2.5 | 1.0 |
| Current collector ($m_{cc}$) | Ti foil ($m_{cc}$) | 4.42 | 2.0 |
| Separator ($m_{sp}$) | GF/A Whatman | 5.3 | 3.0 |
| Electrolyte ($m_{E}$) | 2 M ZnSO­­_4_ aqueous solution (90 *μ*L) | 104 | 56 |

**Table S3.** The relationship among the areal mass loading of active carbon materials ($m_{c}$), positive electrode ($m_{P}$), minimum electrolyte ($m_{E,min}$), and the E/C ratio.

| $\boldsymbol{m}_{\boldsymbol{c}}$  **(mg cm^-2^)** | 2 | 6 | 10 | 14 | 18 | 22 | 26 | 30 | 34 | 38 | 42 | 46 | 50 |
| --- | --- | --- | --- | --- | --- | --- | --- | --- | --- | --- | --- | --- | --- |
| $\boldsymbol{m}_{\boldsymbol{P}}$  (**mg cm^-2^**) | 2.5 | 7.5 | 12.5 | 17.5 | 22.5 | 27.5 | 32.5 | 37.5 | 42.5 | 47.5 | 52.5 | 57.5 | 62.5 |
| $\boldsymbol{m}_{\boldsymbol{E}, \boldsymbol{min}}$  (**mg cm^-2^**) | 3.82 | 7.86 | 11.9 | 15.9 | 19.9 | 24.0 | 28.0 | 32.1 | 36.1 | 40.2 | 44.2 | 48.2 | 52.3 |
| **E/C** | 1.91 | 1.31 | 1.19 | 1.14 | 1.11 | 1.09 | 1.08 | 1.07 | 1.06 | 1.06 | 1.05 | 1.05 | 1.05 |

**Table S4** Areal mass of all components in a representative ZIHC model (the mass of cell casing and other supporting materials are excluded).

| **Cell components** | **Material details** | **Areal Mass**  **(mg cm^-2^)** |
| --- | --- | --- |
| Negative electrode ($m_{N}$) | Zn foil ($m_{Zn}$) | $\frac{\frac{N}{P}\times Q_{g,c}\times m_{c}}{Q_{g,Zn}}$ |
| Positive electrode ($m_{P}$) | Carbon materials ($m_{c}$) | $m_{c}$ |
|  | Conductive additive ($m_{ca}$) | 0.125$m_{c}$ |
|  | Binders ($m_{B}$) | 0.125$m_{c}$ |
| Current collector ($m_{cc}$) | Ti foil ($m_{cc}$) | 4.42 |
| Separator ($m_{sp}$) | Celgard 3501 PP ($m_{sp}$) | 1.4 |
| Electrolyte ($m_{E}$) | 2 M ZnSO­­_4_ aqueous solution (lean electrolyte) ($m_{E,min}$) | $m_{E,c}+m_{E,ca}+m_{E,sp}$ |
|  | 2 M ZnSO­­_4_ aqueous solution (flooding electrolyte) ($m_{E}$), E/C from 2 to 50 | $\frac{E}{C}\times m_{c}$ |

**Calculation of the increasing rate of** $\boldsymbol{E}_{\boldsymbol{g,cell}}$ **over** $\boldsymbol{\Delta}\boldsymbol{m}_{\boldsymbol{c}}$ **(**$\frac{\boldsymbol{\Delta}\boldsymbol{E}_{\boldsymbol{g,cell}}}{\boldsymbol{\Delta}\boldsymbol{m}_{\boldsymbol{c}}}$ **):**

${Q_{A,cell}=Q}_{A,P}=0.001\times Q_{g,c}\times m_{c}$ (S6)

$m_{cell}=\frac{\frac{N}{P}\times Q_{g,c}\times m_{c}}{Q_{g,Zn}}+1.25m_{C}+5.82+\frac{E}{C}\times m_{c}$ (S7)

$E_{g,cell}=\frac{U\times Q_{A,cell}}{{10}^{-3}\times m_{cell}}$ (S8)

$E_{g,cell}$ (**Equation S8**) can be correlated with respect to carbon mass loading ($m_{c}$, mg cm^-2^) (**Equation S9**) by substituting $Q_{A,cell}$ (**Equation S6**) and $m_{cell}$ (**Equation S7**) as follows:

$E_{g,cell}=\frac{U\times Q_{g,c}\times m_{c}}{\frac{\frac{N}{P}\times Q_{g,c}\times m_{c}}{Q_{g,Zn}}+1.25m_{C}+5.82+\frac{E}{C}\times m_{c}}$ (S9)

Given$U=1.6 V$, $Q_{g,c}=50 mAh g^{-1}$, $\frac{N}{P}=2$, $\frac{E}{C}=2$, $Q_{g,Zn}=820 mAh g^{-1}$, the corresponding $E_{g,cell}$ is calculated as follows (**Equation S10**) :

$E_{g,cell}=\frac{80m_{c}}{3.372m_{c}+5.82}$ (S10)

The increasing rate of $E_{g,cell}$ over $\Delta m_{c}$ ($\frac{\Delta E_{g,cell}}{\Delta m_{c}}$, Wh kg^-1^/mg cm^-2^ ) can be computed as the 1st derivative of $E_{g,cell}$ as follows (**Equation S11**):

$\frac{\Delta E_{g,cell}}{\Delta m_{c}}=\frac{9700000}{3\left( 281m_{c}+485 \right)^{2}}$ (S11)

**References cited in SI**

[1] Y. Lu, Z. Li, Z. Bai, H. Mi, C. Ji, H. Pang, C. Yu, J. Qiu, *Nano Energy* **2019**, *66*. <https://doi.org/10.1016/j.nanoen.2019.104132>.

[2] J. Yin, W. Zhang, W. Wang, N. A. Alhebshi, N. Salah, H. N. Alshareef, *Adv. Energy Mater.* **2020**, *10*. <https://doi.org/10.1002/aenm.202001705>.

[3] Z. Zhou, X. Zhou, M. Zhang, S. Mu, Q. Liu, Y. Tang, *Small* **2020**, *16*, e2003174. <https://doi.org/10.1002/smll.202003174>.

[4] X. Shi, H. Zhang, S. Zeng, J. Wang, X. Cao, X. Liu, X. Lu, *ACS Materials Lett.* **2021**, *3*, 1291-1299. <https://doi.org/10.1021/acsmaterialslett.1c00325>.

[5] X. Shi, J. Xie, F. Yang, F. Wang, D. Zheng, X. Cao, Y. Yu, Q. Liu, X. Lu, *Angew. Chem. Int. Ed. Engl.* **2022**, *61*, e202214773. <https://doi.org/10.1002/anie.202214773>.

[6] J. Wang, Y. Huang, X. Han, Z. Li, S. Zhang, M. Zong, *Appl. Surf. Sci.* **2022**, *579*. <https://doi.org/10.1016/j.apsusc.2021.152247>.

[7] Y. Yang, D. Chen, H. Wang, P. Ye, Z. Ping, J. Ning, Y. Zhong, Y. Hu, *Chem. Eng. J.* **2022**, *431*. <https://doi.org/10.1016/j.cej.2021.133250>.

[8] L. Zhao, W. Jian, J. Zhu, X. Zhang, F. Wen, X. Fei, L. Chen, S. Huang, J. Yin, N. R. Chodankar, X. Qiu, W. Zhang, *ACS Appl. Mater. Interfaces* **2022**, *14*, 43431-43441. <https://doi.org/10.1021/acsami.2c13886>.

[9] P. Shang, M. Liu, Y. Mei, Y. Liu, L. Wu, Y. Dong, Z. Zhao, J. Qiu, *Small* **2022**, *18*, e2108057. <https://doi.org/10.1002/smll.202108057>.

[10] K. Shang, Y. Liu, P. Cai, K. Li, Z. Wen, *J. Mater. Chem. A* **2022**, *10*, 6489-6498. <https://doi.org/10.1039/D2TA00202G>.

[11] F. Wei, H. Zhang, X. Hui, Y. Lv, S. Ran, X. Liu, *J. Power Sources* **2023**, *554*. <https://doi.org/10.1016/j.jpowsour.2022.232348>.

[12] Y. Zhang, P. Xie, C. Jiang, Z. Zou, *J. Energy Storage* **2023**, *57*. <https://doi.org/10.1016/j.est.2022.106169>.

[13] H. Liu, D. Tong, L. Peng, L. Huang, Z. Gu, Z. Sheng, F. Zhang, Y. Zhang, Z. Hu, T. Qian, H. Zhu, *ACs Appl. Energy Mater.* **2023**, *6*, 6752-6759. <https://doi.org/10.1021/acsaem.3c00929>.

[14] H. Liu, S. Li, X. Huang, W. Chen, M. Xu, Y. Ren, R. Zhang, Z. Miao, J. Zhu, *Mater. Today Chem.* **2023**, *29*. <https://doi.org/10.1016/j.mtchem.2023.101476>.

[15] J. Yu, X. Jia, J. Peng, B. Meng, Y. Wei, X. Hou, J. Zhao, N. Yang, K. Xie, D. Chu, L. Li, *ACs Appl. Energy Mater.* **2023**, *6*, 2728-2738. <https://doi.org/10.1021/acsaem.2c03311>.

[16] X. Chen, P. Ye, H. Wang, H. Huang, Y. Zhong, Y. Hu, *Adv. Funct. Mater.* **2023**, *33*. <https://doi.org/10.1002/adfm.202212915>.

[17] X. Huang, W. Chen, H. Liu, S. Li, W. Zhang, Y. Ren, R. Zhang, M. Xu, J. Zhu, *J. Energy Storage* **2023**, *72*. <https://doi.org/10.1016/j.est.2023.108794>.

[18] H. Li, P. Su, Q. Liao, Y. Liu, Y. Li, X. Niu, X. Liu, K. Wang, *Small* **2023**, e2304172. <https://doi.org/10.1002/smll.202304172>.

[19] L. Yao, J. Jiang, H. Peng, H. Yang, S. Liu, X. Wen, P. Cai, Y. Zou, H. Zhang, F. Xu, L. Sun, X. Lu, *J. Energy Storage* **2023**, *58*. <https://doi.org/10.1016/j.est.2022.106378>.

[20] H. Li, Q. Liao, Y. Liu, Y. Li, X. Niu, D. Zhang, K. Wang, *Small* **2024**, *20*, e2307184. <https://doi.org/10.1002/smll.202307184>.

[21] H.-X. Li, W.-J. Shi, L.-Y. Liu, X. Zhang, P.-F. Zhang, Q. Wang, Y. Liu, Z.-Y. Wang, J. Dou, *Chem. Eng. J.* **2024**, *487*. <https://doi.org/10.1016/j.cej.2024.150630>.

[22] Z. Yang, X. Chang, H. Mi, Z. Wang, J. Gao, X. Xiao, F. Guo, C. Ji, J. Qiu, *J. Colloid Interface Sci.* **2024**, *658*, 506-517. <https://doi.org/10.1016/j.jcis.2023.12.097>.

[23] Z. Li, D. Chen, Y. An, C. Chen, L. Wu, Z. Chen, Y. Sun, X. Zhang, *Energy Storage Mater.* **2020**, *28*, 307-314. <https://doi.org/10.1016/j.ensm.2020.01.028>.

[24] X. Zhu, F. Guo, Q. Yang, H. Mi, C. Yang, J. Qiu, *J. Power Sources* **2021**, *506*. <https://doi.org/10.1016/j.jpowsour.2021.230224>.

[25] L. Wang, M. Peng, J. Chen, X. Tang, L. Li, T. Hu, K. Yuan, Y. Chen, *ACS Nano* **2022**, *16*, 2877-2888. <https://doi.org/10.1021/acsnano.1c09936>.

[26] Y. Zhang, X. Li, Y. Li, X. Zhang, D. Yu, C. Chen, G. Zhao, *Electrochim. Acta* **2023**, *447*. <https://doi.org/10.1016/j.electacta.2023.142114>.

[27] J. Chen, L. Wang, M. Peng, T. Hu, K. Yuan, Y. Chen, *Chem. Mater.* **2023**, *35*, 4089-4099. <https://doi.org/10.1021/acs.chemmater.3c00563>.
